# The Influence of Physical Fitness on Reasons for Academy Separation in Law Enforcement Recruits

**DOI:** 10.3390/ijerph16030372

**Published:** 2019-01-29

**Authors:** Robert G. Lockie, Katherine Balfany, Ashley M. Bloodgood, Matthew R. Moreno, Karly A. Cesario, Joseph M. Dulla, J. Jay Dawes, Robin M. Orr

**Affiliations:** 1 Department of Kinesiology, California State University, Fullerton, Fullerton, CA 92831, USA; kbalfany@csu.fullerton.edu (K.B.); abloodgood17@csu.fullerton.edu (A.M.B.); moreno.matthewr@csu.fullerton.edu (M.R.M.); cesariokarly@csu.fullerton.edu (K.A.C.); 2 Recruit Training Unit, Training Bureau, Los Angeles County Sheriff’s Department, Los Angeles, CA 90022, USA; JMDulla@lasd.org; 3 Department of Health Sciences, University of Colorado-Colorado Springs, Colorado Springs, CO 80918, USA; jdawes@uccs.edu; 4 Tactical Research Unit, Bond University, Robina 4229, QLD, Australia; rorr@bond.edu.au

**Keywords:** aerobic capacity, attrition, change-of-direction speed, deputy sheriff, graduation, high-intensity running, police, strength endurance, tactical

## Abstract

This study analyzed the effects physical fitness may have on reasons for academy separation in law enforcement recruits. A retrospective analysis was conducted on 401 recruits; 330 recruits graduated (GRAD), and 71 recruits separated at various times during academy. Twenty-eight recruits separated for personal reasons (SEPPR); 18 due to physical training failures (i.e., poor fitness) or injury (SEPFI); and 25 due to academic or scenario failures (SEPAS). Fitness testing occurred prior to academy, and included: Push-ups and sit-ups in 60s; a 75-yard pursuit run (75PR); vertical jump; medicine ball throw; and multistage fitness test (MSFT). A one-way ANOVA with Bonferroni post hoc compared between-group fitness test performance. A multiple stepwise regression calculated whether recruit characteristics or fitness could predict separation. The GRAD group was younger than the SEPAS group (*p* < 0.01), faster in the 75PR than the SEPFI group (*p* = 0.02), and completed more MSFT shuttles than the SEPPR and SEPFI groups (*p* = 0.01). Age predicted GRAD and SEPAS group inclusion; MSFT predicted GRAD, SEPPR, and SEPFI group inclusion. Recruits who had superior high-intensity running capacity (75PR) and aerobic fitness (MSFT) should have a better chance of completing academy. However, this could be influenced by training practices adopted during academy.

## 1. Introduction

Law enforcement can be a demanding profession that can place high levels of physical [1] and psychological [2,3] stress on those employed in this vocation. The academy period is used by law enforcement academy (LEA) instructors and tactical strength and conditioning facilitators (TSAC-F) to train recruits to tolerate the physical and psychological challenges of policing, while also teaching the necessary procedures and skills required for the job [4,5,6]. However, not all recruits will graduate from academy. Recruits may separate (i.e., they do not graduate) for a number of different reasons. These reasons may include personal reasons (e.g., they no longer want to work in law enforcement) [3], physical training (PT) session failures (i.e., they do not complete the requisite number of sessions as mandated by the LEA or state) [7], injury [5,8,9], failure in academics or scenario-based training [10,11,12]. Recruits that separate create a significant financial burden to an agency [5,13]. Thus, it would be pertinent for agencies to understand whether there are certain physical characteristics that influence whether a recruit graduates or separates from academy. If these characteristics could be effectively measured prior to academy, it may provide useful information for an agency to make more cost-effective decisions as to whether they hire certain individuals.

A major component of academy is PT, which should be tailored towards developing recruits such that they can complete the tasks required in law enforcement. Greater aerobic fitness (as measured via number of shuttles in the 20-m multistage fitness test; MSFT) and strength endurance (e.g., push-up and sit-up repetitions) have been correlated with better performance in job-specific tasks [6,12], which highlights the need for fitness in law enforcement populations. Specific to academy graduation, Shusko et al. [13] found that Massachusetts-based recruits in the USA who completed fewer push-ups in 60 s prior to academy, and had a slower 2.4-km run time, were more likely to separate. Orr et al. [9] found that lower-body power measured via vertical jump (VJ) performance was a predictor of injury or illness in Australian police recruits. Accordingly, physical fitness would likely be a factor influencing a recruit’s ability to successfully fulfill the requirements of academy and graduate.

A limitation with PT in the academy setting is that agencies may lack the equipment and space to conduct a variety of training practices (i.e., maximal strength training) which could be useful for a law enforcement officer (LEO) [6]. As a result, many academies tend to focus on strength endurance or callisthenic-type exercises conducted in a circuit training fashion [14], in addition to aerobic-focused training (e.g., long, slow distance or formation runs) [15]. These practices are often conducted within a paramilitary ‘one-size-fits-all’ training model [5,6,8,14,15]. This style of training may lead to an inappropriate application of training load for certain recruits, which could then increase their risk of injury and/or separation [5,16]. The PT practices adopted by agencies may place greater importance on certain physical qualities for recruits (i.e., if running is a focus, then aerobic fitness may be more important for a recruit). This should also be considered when analyzing the physical fitness qualities influencing academy graduation in LEA recruits.

Academy training is used not just to physically develop recruits, but also to challenge them psychologically in order to assess each recruit’s tolerance and demeanor under stress [3]. Berg [2] noted that the stress imposed by the verbal commands of training staff is generally designed to test the mental toughness and resilience of recruits, and find any character flaws that may impede being an effective LEO. As an example, a recruit that displays poor emotional stability in the face of adversity during academy may not be the best candidate to become a LEO, where there are major consequences to poor decisions made in the field under stress. Notably, higher physical and psychological stress has been associated with thoughts about quitting the profession in correctional officers [17]. Recruits with lower levels of fitness may not only find the PT more difficult, but could also experience greater psychological stress within the academy environment. Noting the general adaptation of the biological system to stress proposed by Selye [18], the combination of stress imparted by both physical and psychological stressors may lead to system exhaustion and in turn impact on motivation. Similarly, academic stress can have the same effect when combined with physical stress. As an example, in collegiate athletes, the risk of injury was found to higher during periods of high academic stress when compared to periods of lower academic stress [19]. To the author’s knowledge, there has been no analysis of the relationship between physical fitness and voluntary decisions made by recruits to separate from a law enforcement training academy, nor regarding any relationships between physical fitness and academic failure during training academy.

Therefore, the purpose of this retrospective study was to analyze the effects physical fitness may have on academy graduation and reasons for separation in LEA recruits. Although there are limitations with conducting retrospective analyses, this is often a necessity in law enforcement research, due to the constraints and external demands placed on these populations. Furthermore, this is very common in the scientific literature [1,4,6,12,13,20,21,22,23,24,25,26,27,28,29,30,31,32,33,34]. For this study, the recruits who did not graduate were divided into groups according to whether they: Separated for personal reasons; separated due to PT failures (i.e., poor fitness) or injury; or separated due to academic or scenario failures. It was hypothesized that recruits who graduated would display superior physical fitness across the different assessments utilized in this study. This would occur regardless of the reason why a recruit may have separated.

## 2. Materials and Methods

### 2.1. Subjects

Retrospective analysis of five academy classes from one agency was conducted. This sample was comprised of 401 recruits (age: 27.30 ± 5.92 years; height: 1.74 ± 0.12 m; body mass: 80.27 ± 14.38 kg), which included 333 males (age: 27.31 ± 5.99 years; height: 1.76 ± 0.12 m; body mass: 83.26 ± 12.66 kg) and 68 females (age: 27.24 ± 5.66 years; height: 1.64 ± 0.07 m; body mass: 66.45 ± 13.89 kg). The five training cohorts started their academy within a calendar year in southern California. Any strength and conditioning or training programs prior to academy were generally completed voluntarily at the individual-level only by recruits [23,27]. Based on the archival nature of this analysis, the institutional ethics committee approved the use of pre-existing data (HSR-17-18-370).

### 2.2. Procedures

The data in this study were collected by staff working for one LEA, and the procedures have been detailed in the literature [23,27]. While tests of other physical capacities (e.g., flexibility, linear speed, strength, etc.) would have been beneficial to include, this was not possible given the confines of time, equipment, and logistical restrictions provided by the LEA. Nonetheless, the fitness capacities that were assessed within this study are typical of law enforcement recruits in the literature [6,20,23,26,27,28]. The staff (~20 per testing session) were all trained by a certified TSAC-F who verified the proficiency of the staff members before each session, and all staff followed strict instructions (which will be detailed) to conduct each test. Each recruit’s age, height, and body mass were recorded at the start of academy training. Height was measured barefoot using a portable stadiometer (seca, Hamburg, Germany), while body mass was recorded by electronic digital scales (Health o Meter, Neosho, Missouri). As detailed by Lockie et al. [27], all tests were conducted outdoors on concrete or asphalt surfaces at the LEA’s training facility on a day scheduled by the staff for the LEA. Testing typically occurred between the hours of 09:00–14:00 depending on recruit availability, and recruits generally did not eat in the 2–3 h prior to their testing session as they were completing non-strenuous activity and employee-specific documentation for the LEA. The weather conditions for testing were typical of the climate of southern California during a calendar year. Although conducting testing outdoors is not ideal, there was no indoor testing facility available for this LEA and these procedures were typical of staff from the LEA [6,27]. Recruits rotated through the assessments in small groups of 3–4 and were permitted to consume water as required during the testing session.

### 2.3. Push-Ups

Upper-body muscular endurance was assessed via a maximal push-up test where recruits completed as many repetitions as possible in 60 s. The protocol for this assessment followed that of established law enforcement research, where a tester placed a fist on the floor directly under the recruit’s chest to ensure they descended to an appropriate depth [1,4,6,22,24,26,27,30,31,33,35]. All female recruits were partnered with a female tester. Recruits started in the standard ‘up’ position, with the body taut and straight, the hands positioned shoulder-width apart, and the fingers pointed forwards. On the start command, a tester began the stopwatch, and recruits flexed their elbows, lowered themselves until their chests contacted another tester’s fist, before extending their elbows to return to the start position. The recruits performed as many push-ups as possible in 60 s using this technique.

### 2.4. Sit-Ups

Abdominal muscular endurance was assessed via the sit-up test, where recruits completed as many repetitions as possible in 60 s. The sit-up test was conducted according to procedures established in previous law enforcement research [1,4,6,20,22,24,26,27,30,31,36,37]. Recruits laid on their backs with their knees flexed to 90°, heels flat on the ground, and arms crossed across their chest and hands positioned on their opposing shoulders. The feet were held to the ground by a tester. On the start command, recruits raised their shoulders from the ground while keeping their arms crossed, and touched their elbows to their knees. They then descended back down until the shoulder blades contacted the ground. Recruits completed as many repetitions as possible with this technique in the 60-s time period.

### 2.5. 75-Yard Pursuit Run (75PR)

**Figure 1:**
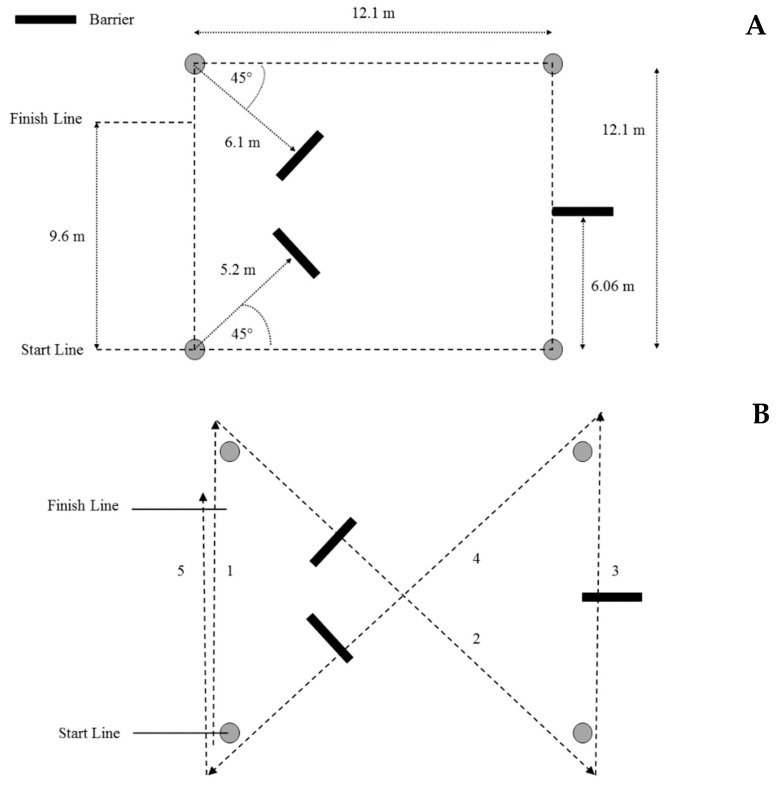
(**A**) The dimensions for the 75-yard pursuit run (75PR) in meters and (**B**) the running direction (numbered in order) for the 75PR.

The 75-yard (68.58-meter) pursuit run (75PR) was designed to simulate a foot pursuit for a LEO [20,27,28], and is shown in Figure 1. Although yards is an Imperial measure, the 75PR is the standard name for this test [38], has been used in the literature [20,27,28], and will be used in this study for clarity. The recruit completed five linear sprints about a square grid (each side was 12.1 m), while completing four, 45° direction changes zig-zagging across the grid. Recruits were also required to step over three barriers that were 2.44 m long and 0.15 m high that simulated road-side curbs during three of the five sprints. Time was recorded via a stopwatch, from the initiation of movement at the start, until the recruit crossed the finish line. Timing via stopwatches is standard practice in LEA testing [1,4,20,22,26,27,28,29,30,37,39]. Furthermore, testers trained in the use of stopwatch timing procedures for running tests can record reliable data [40,41].

### 2.6. Vertical Jump (VJ)

A Vertec apparatus (Perform Better, Rhode Island, USA) was used to measure the VJ, and followed established assessment protocols [4,23,30,37,42,43]. The recruit initially stood side-on to the Vertec (on the recruit’s dominant side), reached upward as high as possible, and while keeping their heels on the ground, fully elevated the shoulder to displace as many vanes as possible. The last vane moved became the zero reference. The recruit then jumped as high as possible with no preparatory step, and tapped the highest vane they could with their dominant hand. Height was recorded from highest vane moved. No restrictions were placed on the range of countermovement during the jump. VJ height was calculated in inches by subtracting the standing reach height from the jump height, before being converted to cm [23,30]. Each recruit completed two trials, with a recovery time between trials of approximately 30–60 s, and the best trial used for analysis.

### 2.7. Medicine Ball Throw (MBT)

The MBT was used to indirectly measure upper-body power, and the procedures were adapted from the literature [23,27,44]. Recruits sat on the ground with their head, shoulders, and lower back against a concrete wall, and projected a 2-kg medicine ball (Champion Barbell, Texas, USA), which was lightly dusted with chalk, as far as possible using a two-handed chest pass. The measurement taken, using a standard tape measure, was the perpendicular distance from the wall to the chalk-marking closest to the wall made by the ball [23,44,45]. Two trials were completed, with a recovery time between trials of approximately 30–60 s, and the best trial was used for analysis.

### 2.8. 20-m Multistage Fitness Test (MSFT)

The MSFT was used to measure maximal aerobic capacity in the recruits, and followed established procedures [12,27,46]. Recruits were required to run back and forth between two lines spaced exactly 20 m apart, which were indicated by markers. The speed of running for this test was standardized by pre-recorded auditory cues (i.e., beeps) played from an iPad handheld device (Apple Inc., Cupertino, California, USA) connected via Bluetooth to a portable speaker (ION Block Rocker, Cumberland, Rhode Island, USA). The speaker was located in the center of the running area, and positioned in such that it would not interfere with the recruits. The test was terminated when the recruit was unable to reach the lines twice in a row in accordance with the auditory cues. This test was scored according to the final stage the recruit was able to achieve, and the stage was used to calculate the total number of completed shuttles.

### 2.9. Statistical Analysis

Information as to whether recruits completed academy and graduated, or did not and were separated, were provided by training staff from the LEA. Recruits were then split into four groups based on the information provided by LEA staff: Those that graduated (GRAD), and those that separated for personal reasons (SEPPR), PT failures or injury (SEPFI), or due to academic or scenario failures (SEPAS). Separation due to PT failures and injury were initially two separate groups. However, they were combined into one group, due to less fit recruits being more likely to get injured during academy [47], and because injuries often led to a recruit not completing the required number of PT sessions (which then resulted in academy separation). Sexes were combined within these groups, as all recruits need to attain the same standards to graduate academy, regardless of sex. This approach has been used in previous research [20,22,23,27,28].

Statistical analyses were computed using the Statistics Package for Social Sciences (Version 25.0; IBM Corporation, New York, USA) and Microsoft Excel (Microsoft Office Professional Plus 2016, Microsoft Corporation, Washington, WA, USA). Descriptive statistics (mean ± standard deviation [SD]) were calculated for each test parameter. A one-way analysis of variance (ANOVA), with Bonferroni post hoc for multiple comparisons, was used to calculate any performance differences in the fitness tests between the four groups. Significance was set at *p* < 0.05 a priori. Similar to previous research [23], effect sizes (*d*) were also calculated for the between-group comparisons, where the difference between the means was divided by the pooled SD [48]. In accordance with Hopkins [49], a *d* less than 0.2 was considered a trivial effect; 0.2 to 0.6 a small effect; 0.6 to 1.2 a moderate effect; 1.2 to 2.0 a large effect; 2.0 to 4.0 a very large effect; and 4.0 and above an extremely large effect. Multiple stepwise linear regression was used to determine whether age, height, body mass, or the physical fitness tests predicted graduation or reasons for separation in the recruits. As group inclusion was a categorical variable within SPSS, the data were recoded into dummy variables to provide dichotomous values (1 = group inclusion for either GRAD, SEPPR, SEPFI, or SEPAS; 0 = all other groups). Thus, GRAD, SEPPR, SEPFI, or SEPAS each acted as a dependent variable [6].

## 3. Results

Across the five classes, 330 recruits graduated (GRAD), while 71 recruits separated at various time points during academy. Of these, 28 recruits were placed in the SEPPR group, 18 in the SEPFI group, and 25 in the SEPAS group. Table 1 displays the descriptive data for all groups, while Table 2 shows the effect size data for the pairwise comparisons. The GRAD group was significantly younger than the SEPAS (*p* < 0.01) group, which had a moderate effect. With regards to the 75PR, the GRAD group was significantly faster than the SEPFI (*p* = 0.02) group, and this had a moderate effect. The GRAD group also completed significantly more MSFT shuttles than the SEPPR and SEPFI groups (both *p* = 0.01 with moderate effects). There were no significant between-group differences for height, body mass, the push-up and sit-up assessments, VJ, or MBT.

The multiple stepwise linear regression data is shown in Table 3. Age and the MSFT predicted inclusion in the GRAD group, with 9% explained variance. The MSFT predicted inclusion in the SEPPR and SEPFI group, with 1–2% explained variance. Age predicted inclusion in the SEPAS group, with 5% explained variance.

## 4. Discussion

This study documented the differences in physical fitness between recruits from one LEA who graduated or separated from academy for a variety of reasons. The results provided some support to the study hypotheses. Recruits that graduated were faster in the 75PR and completed more MSFT shuttles when compared to recruits who separated, due to PT failures or injury. Graduating recruits also completed more MSFT shuttles than recruits who separated for personal reasons. However, there were no differences between any of the groups for performance in the push-up, sit-up, VJ, and MBT assessments. These data suggest that, for this agency, there are specific fitness characteristics that could influence and predict whether a recruit graduates academy. Although the stepwise regression data tended to have very low explained variance, it did tend to support the between-group comparison data. It should however be noted that, while the data exhibited a low albeit significant variance, the training stimulus provided during academy occurs across multiple occasions, rather than a single one-off event. As such, there is essentially a cumulative impact of risk. This phenomenon of differential risk accrual over repeated exposures to events occurring during the training program, is discussed in detail by Pope [50]. As will be also be discussed, this could be influenced by the PT practices adopted by the LEA.

The age, height, and body mass of the recruits in this study were typical of that established in the literature [6,20,23,27,28]. Height and body mass were not significantly different between any of the groups. However, the recruits in the SEPAS group were significantly older than the GRAD group, and age was a predictor of inclusion in this group. Age was also a predictor for inclusion in the GRAD group. Recruits in the SEPAS group either failed academic examinations or the requirements needed in law enforcement-specific training scenarios. Time management [51], and perceived control of time management [52], is important for academic success. Recruits in their late 20’s and early 30’s may have more outside life influences (e.g., family commitments) that could have influenced the results seen in this study for the SEPAS recruits. Noting the trend toward lower MSFT and slower 75PR results, older officers also tend to have lower levels of physical fitness when compared to their younger counterparts [22,23,31]. This could have influenced the recruits in the SEPAS groups’ ability to adequately recover from the stressors of academy, and their subsequent performance in training scenarios. These notions, however, cannot be confirmed by the current research. Nonetheless, outside time commitments and differences in fitness for older recruits could play some role in their ability to complete the academic and scenario training requirements of academy, and could be points to consider for LEA staff.

The SEPFI group performed poorer in the two running assessments (75PR and MSFT) compared to the GRAD group, and the MSFT (albeit with a low explained variance) also predicted inclusion in this group. The 75PR measures high-intensity running and change-of-direction speed [27,28], while the MSFT provides a valid measure of aerobic capacity [53,54]. Running is a heavy focus of PT in the academy period for this LEA [15], so it is understandable that recruits who were superior in running tasks would be in a better position to complete the academy PT requirements and graduate. Furthermore, fitter recruits tend to operate at a lower percentage of their maximal capacity, and as a result can perform certain tasks for longer, fatigue less rapidly, and recover more quickly [55,56]. Shusko et al. [13] also found that police recruits who had a slower 2.4-km run time, which is indicative of lower aerobic fitness, were more likely to separate from academy. Recruits attending academy with poorer levels of fitness, as measured via running tasks, such as the 75PR and MSFT, could be more likely to separate. This could be especially true if a traditional LEA training model, with an emphasis on strength endurance and aerobic fitness, is followed [14,15].

The SEPPR recruits also completed fewer MSFT shuttles than the GRAD group, and the MSFT was a predictor (with a low explained variance) for inclusion in the SEPPR group. A factor that could influence whether a recruit separates for personal reasons is the inappropriate application of PT. As noted, many LEA academies operate via a paramilitary, ‘one-size-fits-all’ model [5,6,8,14,15], where every recruit is expected to complete the same training regardless of their current fitness or ability. Additionally and as noted, traditional LEA training can often involve a high volume of running [15]. Recruits in the SEPPR group may have found the training adopted by the LEA staff beyond their current physical capacity, which could then have contributed to their voluntary decision to separate. There are certain physiological characteristics that can predispose an individual to be a better runner (e.g., maximal aerobic capacity, lactate threshold, and running economy) [57,58]. While these qualities are trainable [57], there will always be recruits who are better suited to running than others (e.g., they have superior genetics relative to aerobic capacity or running biomechanics) [59,60]. LEA training staff should be wary that they do not lose recruits who are potentially competent LEOs that can perform the relevant job tasks, but are not good distance (e.g., 800 m or longer) runners. Physical fitness can still be improved with lower volumes of running in law enforcement populations, with a concurrent decrease in the risk of injury [5,8]. Given the findings of Trank et al. [61], whereby Naval recruits who ran <40 km (25 miles) where less likely to be injured than those who ran >40 km without negatively affecting physical readiness, future research should investigate moderating the volume and mileage of running during a law enforcement academy, and whether this can influence graduation and separation rates.

It is possible that certain recruits from the SEPPR group resigned for reasons not related to their physical fitness. Further to the PT requirements, a major part of academy is the development of the inherent discipline required in the profession. This can be imposed by the training staff offering very stern commands and directions for expected behaviours [2,3]. Although not measured in this study, there are certain personality types who would be more likely to voluntarily resign for personal reasons. Individuals that get more emotionally upset, and are more tender-minded, carefree, and impulsive, are more likely to voluntarily terminate employment as a police officer [62]. Given the high psychological stress imposed by training officers during academy [2,3], some recruits may have voluntarily resigned due to this stress, as opposed to that from PT. While this is a possibility, it is important to note that, due to the nature of academy, the psychological and physical stressors imposed by staff will be experienced simultaneously. Further, the recruits in the SEPPR group still did perform worse in the MSFT compared to the GRAD group. If a recruit had personality traits that did not fit well with the law enforcement profession, this could be compounded by poorer aerobic fitness, especially if PT had a high volume of running [15]. Future research should investigate the relationships between reasons for separation and personality traits, and other psychological variables (e.g., self-efficacy, motivation) that may influence academy separation.

Strength endurance tests, such as push-ups and sit-ups, predominate in the fitness assessment of law enforcement populations [1,4,6,13,20,22,24,26,27,30,31,33,35,36,37]. However, these assessments did not differentiate between recruits who graduated or separated for any reason, or predict any reasons for separation. This is contrary to findings from Shusko et al. [13], who found that recruits performing fewer push-ups tended to separate from LEA academies. The VJ and MBT also did not differentiate or predict recruits who graduated or separated. This contrasts with Orr et al. [9], who found poorer VJ performance was a predictor of injury or illness in Australian police recruits. Injury or illness can lead to academy separation for a recruit [5,8,9], especially if they miss a number of PT sessions [7]. However, the use of body weight calisthenics [14] and the volume of running [15] completed during PT at this academy could have limited the impact upper- or lower-body power may have had on recruit graduation rates. These results emphasize that the PT model adopted by LEA training staff will influence those fitness characteristics that could predispose a recruit to graduating or separating from academy.

There are certain limitations to this study that should be noted. This study incorporated a pre-existing fitness testing battery specific to one LEA. Different agencies may use different assessments (e.g., the 2.4-km run instead of the MSFT) [1,12,20,29,30,31,63]. This could influence the effects fitness may demonstrate with academy graduation rates. The study results could have also been influenced by the nature of the MSFT, where recruits can voluntarily terminate the test. Training staff could not always guarantee that all recruits reached their maximal aerobic capacity. Nonetheless, this test is an established and valid measure of maximal aerobic capacity [54,64,65], and the data from the recruits in this study was similar to that from previous law enforcement research [27]. Due to the sample size, recruits with PT failures and injuries were grouped together. Even though less fit recruits are more likely to get injured because the training load could exceed their capacities [47], it is still possible that certain injuries may have been suffered by recruits with higher levels of fitness. No maximal strength tests were included in the battery used in this study, although this is typical of law enforcement research [6,12,20,22,23,24,26,27,28,33]. Future research should investigate whether maximal strength could influence whether a recruit graduates or separates from academy. It would have been beneficial to monitor recruit fitness at multiple time points throughout academy. However, timetables can vary across different academy classes, due to the wide variety of skills and procedures that need to be taught to recruits [4,5,6], and ensuring the requisite staff are available to instruct. Nonetheless, this should be explored in future research.

## 5. Conclusions

The results from this study indicated that there were certain fitness characteristics that could influence reasons for academy separation in law enforcement recruits. Recruits that failed academic or training scenarios tended to be older than recruits who graduated. This could be influenced by time commitments external to academy for older recruits, or differences in fitness levels when compared to their younger counterparts that could influence recovery from academy stress. High-intensity running and change-of-direction speed, as measured by the 75PR, and aerobic fitness indicated by the MSFT, were poorer in recruits that separated, due to PT failures or injury. Recruits that separated for personal reasons also had lower aerobic fitness as measured by the MSFT. On the surface, these data suggest that LEA recruits should enhance their high-intensity running and change-of-direction speed (including strength, power, and sprint ability) and aerobic fitness prior to academy to improve their chances of successful graduation. However, these results could also have been influenced by the PT practices adopted by the LEA staff, which involved high volumes of running. Recruits should be aware of the PT training adopted by a LEA, so that they can best prepare for the rigors of that specific academy. Additionally, LEA training staff should consider the total training load they impose during academy to ensure it is not beyond the physical capabilities of some recruits, as this could contribute to injuries or negatively affect a recruit’s motivation to graduate.

## Figures and Tables

**Table 1:** Descriptive data (mean ± SD) for age, height, body mass, and fitness test performance data for law enforcement academy (LEA) recruits who graduated (GRAD) or separated (SEPPR, SEPFI, and SEPAS) from academy training.

	GRAD (*n* = 330)	SEPPR (*n* = 28)	SEPFI (*n* = 18)	SEPAS (*n* = 25)
Age (years)	26.67 ± 5.19	29.35 ± 8.02	29.59 ± 6.88	32.70 ± 9.01 *
Height (m)	1.75 ± 0.09	1.74 ± 0.10	1.72 ± 0.07	1.74 ± 0.08
Body Mass (kg)	80.69 ± 14.38	74.57 ± 14.52	79.50 ± 16.27	80.00 ± 12.54
Push-ups (no.)	42.48 ± 15.09	39.48 ± 14.01	34.63 ± 16.44	40.38 ± 12.24
Sit-ups (no.)	36.19 ± 9.04	35.78 ± 8.72	33.13 ± 7.59	34.29 ± 10.39
75PR (s)	16.97 ± 1.32	17.60 ± 1.21	17.94 ± 1.37 *	17.69 ± 1.28
VJ (cm)	53.60 ± 12.53	51.58 ± 13.43	47.94 ± 11.69	53.34 ± 11.83
MBT (m)	5.84 ± 1.22	5.52 ± 1.35	5.73 ± 1.29	5.96 ± 1.01
MSFT shuttles (no.)	52.75 ± 16.69	41.54 ± 10.74 *	39.94 ± 13.03 *	46.08 ± 11.19

* Significantly (*p* < 0.05) different from the GRAD group. GRAD, graduated; SEPPR, separated for personal reasons; SEPFI, separated for physical training failures or injury; SEPAS, separated for academic or scenario failures; 75PR, 75-yard pursuit run; VJ, Vertical Jump; MBT, Medicine Ball Throw; MSFT, multistage fitness test.

**Table 2:** Pairwise effect size data between LEA recruits who graduated (GRAD) or separated (SEPPR, SEPFI, and SEPAS) from academy training for age, height, body mass, number of push-ups and sit-ups completed in 60 s, time to complete the 75PR, VJ, MBT, and MSFT shuttles.

	GRAD-SEPPR	GRAD-SEPFI	GRAD-SEPAS	SEPPR-SEPFI	SEPPR-SEPAS	SEPFI-SEPAS
Age	0.40	0.48	0.82 *	0.03	0.39	0.39
Height	0.11	0.37	0.12	0.23	<0.01	0.27
Body Mass	0.42	0.08	0.05	0.32	0.40	0.03
Push-ups	0.21	0.50	0.15	0.32	0.07	0.40
Sit-ups	0.05	0.37	0.20	0.32	0.16	0.13
75PR	0.50	0.72 *	0.55	0.26	0.07	0.19
VJ	0.16	0.47	0.02	0.29	0.14	0.46
MBT	0.25	0.09	0.11	0.16	0.37	0.20
MSFT shuttles	0.80 *	0.86 *	0.47	0.13	0.41	0.54

* Moderate effect for the pairwise comparison.

**Table 3:** Stepwise linear regression analysis for inclusion in each group (GRAD, SEPPR, SEPFI, and SEPAS) and age, height, body mass, push-ups, sit-ups, 75PR, VJ, MBT, and MSFT.

Variables	*r*	*r* ^2^	Adjusted *r*^2^
GRAD			
Age	0.263	0.069	0.067
Age, MSFT	0.310	0.096	0.091
SEPPR			
MSFT	0.129	0.017	0.014
SEPFI			
MSFT	0.143	0.020	0.017
SEPAS			
Age	0.223	0.050	0.047
